# Access to veterinary care: evaluating working definitions, barriers, and implications for animal welfare

**DOI:** 10.3389/fvets.2024.1335410

**Published:** 2024-01-18

**Authors:** Kayla Pasteur, Alessia Diana, Jane Kinkus Yatcilla, Shanis Barnard, Candace C. Croney

**Affiliations:** ^1^Department of Comparative Pathobiology, Purdue University College of Veterinary Medicine, West Lafayette, IN, United States; ^2^Purdue University Libraries, Purdue University, West Lafayette, IN, United States; ^3^Center for Animal Welfare Science, Departments of Comparative Pathobiology and Animal Science, Purdue University, West Lafayette, IN, United States

**Keywords:** animal health services, well-being, companion animals, livestock animals, stakeholders, demographics, resources

## Abstract

Humans have a moral obligation to meet the physical and mental needs of the animals in their care. This requires access to resources such as veterinary care, which is integral to achieving animal welfare. However, “access” to veterinary care is not always homogenous across communities and currently lacks a consistent definition. The objectives of this scoping review were to (1) understand how “access” to veterinary care has been defined in the literature, (2) map a broad list of potential barriers that may influence access to veterinary care, and (3) identify how access to care impacts the welfare of companion and livestock animals. The literature search yielded a total of 1,044 publications, 77 of which were relevant to our inclusion criteria, and were published between 2002 and 2022. Studies were most frequently conducted in the United States (*n* = 17) and Canada (*n* = 11). Publications defining access to veterinary care (*n* = 10) or discussing its impacts on animal welfare (*n* = 13) were minimal. However, barriers to accessing veterinary care were thoroughly discussed in the literature (*n* = 69) and were categorized into ten themes according to common challenges and keywords, with financial limitations (*n* = 57), geographic location (*n* = 35), and limited personnel/equipment (*n* = 32) being the most frequently reported. The results of this scoping review informed our proposed definition of access to veterinary care. Additionally, our findings identified a need to further investigate several understudied barriers relating to access to care (i.e., veterinarian-client relationship, client identity) and to better understand how they potentially affect animal welfare outcomes.

## 1 Introduction

Over thousands of years of domestication, humans have developed close bonds with the animals in our care. Domestic species have become intertwined in nearly every aspect of our lives, with livestock often serving as sources of transportation, income, or food. Additionally, perceptions of many companion species have evolved from existing primarily for utility to being cherished family members across the globe (1, 2). Several studies have demonstrated that interacting with companion animals can result in multiple physiological and psychological benefits (3, 4) although some studies have also reported contradicting results [see (5)]. The overall quality of the human-animal relationship has been observed to affect the well-being of both the human and animal. For example, some studies have reported a link between the well-being of farmers and that of the livestock they care for (6, 7). Further, other studies have indicated that the health and well-being of owners can be impacted, especially if they are closely bonded with their pets, as under certain circumstances some people prioritize the needs of their companion animals at the expense of their own (8, 9).

Livestock and companion animals are sentient beings and the species in our care heavily rely upon humans for their survival and well-being. This suggests that humans have an ethical responsibility, or moral obligation, to reduce unnecessary suffering and meet the physical and mental needs of the animals in their care. Meeting these needs requires access to resources such as veterinary care, which is integral to achieving and maintaining animal welfare. However, access to veterinary care is not always homogenous across communities due to barriers such as financial limitations, lack of transportation, limited service providers or proximity to one, and even cultural barriers that may contribute to potential disparities (10, 11). Many pet-owners and livestock producers, hereafter referred to as animal caretakers, rely heavily upon the veterinary community as a resource for both animal care services and animal welfare information (12–14). Therefore, limited access to veterinary care could potentially result in compromised animal welfare outcomes. Studies have reported that a lack of access to veterinary care may result in more frequent outbreaks of disease, increased economic loss, and poor understanding of best management practices (7, 15, 16). Inability to access veterinary care has been suggested to be one of the most significant animal welfare crises in the United States, presenting considerable problems for the health of livestock and companion animals (17–19). Due to these challenges, several groups, including animal welfare organizations, scientific and veterinary communities, as well as members of the public have expressed concerns about how to improve access to veterinary care (20–22). One approach is to analyze and minimize the barriers limiting or preventing access.

Even though some barriers (e.g., socio-economic status, proximity to a provider) have been thoroughly discussed in the literature (19, 23–25), broader understanding of factors that constrain veterinary care, especially in developing countries, is needed along with practical ways to address these challenges. Sparks et al. (11) note that when veterinary and animal welfare organizations deliberately removed structural barriers, individuals were more likely to utilize and benefit from veterinary services. Research discussing access to veterinary care has been conducted for over two decades (26). In that time, some researchers have proposed definitions of ‘access to veterinary care’ which include “Recognizing when a pet needs care, having a veterinary service provider that is physically reachable, and being able to pay for the care.” (19) and “…geographical proximity of resources and service; accessibility of professionals and ease of contact” (27). Yet, the literature has failed to establish a consensus definition of ‘access to veterinary care’. There is also limited information available on many of the factors that potentially influence access to veterinary care and public perceptions on the subject. Further, there is a need to address the potential mismatch in perceptions of access to care between major stakeholders such as the animal sheltering, scientific, and veterinary communities, and animal caretakers, in addition to the public.

For these reasons, a scoping review was conducted to highlight gaps within the literature as a first step toward improving access to veterinary care. The objectives of this scoping review were to (1) build an understanding of how “access” to veterinary care has been broadly defined in the literature to date, (2) provide a map of potential barriers to accessing veterinary care and the extent to which they are discussed in the literature, and (3) identify how access to veterinary care, or lack thereof, may affect the welfare of both companion and livestock animals. We hypothesized that (1) the definition of access to veterinary care would vary amongst different stakeholders (e.g., veterinarians, animal welfare organizations, animal caretakers), and (2) access to veterinary care would differ by region, socioeconomic status, and age.

## 2 Methods

### 2.1 Protocol

An unpublished protocol was prepared under the guidance of an information specialist (author JY) and is available in the Supplementary materials. This review was also written following the Preferred Reporting Items for Systematic Reviews and Meta-Analyzes extension for Scoping Reviews (PRISMA-SCR) (28).

### 2.2 Eligibility criteria

Any publication that reported data on how access to veterinary care is defined, the perceived barriers surrounding access to veterinary care, or its impacts on the welfare of companion or livestock animals were included in this scoping review. Publications where the target animals were studied in shelters, kennels, laboratories, or zoos were excluded to prioritize the investigation of experiences held by individuals, rather than organizations, who are directly involved in the ownership or production of the animals. There were no geographical or date restrictions placed upon the included publications. However, only studies published in English were considered due to resource limitations. Gray literature, or information not controlled by commercial publishers (29), was not considered in this scoping review as we aimed to characterize to what extent published scientific literature has defined ‘access’ to veterinary care and therefore only peer-reviewed publications where the full text was available were included.

### 2.3 Information sources and search

Literature search strategies were composed using index terms and key words to express the concepts of veterinary services, barriers to access, and animal health or welfare. The searches were tailored for the databases CAB Abstracts (Web of Science Platform), PubMed, and Web of Science Core Collection. The searches were executed on December 15, 2022 and 1,269 total results were uploaded to an EndNote library. Duplicates were removed following an iterative method described by Bramer and colleagues (30), and 1,044 results were uploaded to a project on the Covidence screening platform (covidence.org). Two additional duplicates were identified during the full text screening process and were manually removed. The complete search strategies are available in Table 1.

**Table 1:** Search strategies (including index terms and keywords) utilized for the CAB Abstracts, PubMed, and Web of Science Core Collection databases.

Web of Science Core Collection	
Step	Search string
#1	TS = (veterinary NEAR/2 (care OR service* OR healthcare OR treatment*))
#2	TS = (access* OR barrier* OR motivat* OR facilitat* OR challenge*)
#3	TS = (animal* NEAR/3 (welfare OR health OR wellbeing OR “well being”))
#4	#1 AND #2 AND #3
#5	#4 AND LANGUAGE = ENGLISH

PubMed	
#1	“veterinary care”[Title/Abstract:~2] OR “veterinary service”[Title/Abstract:~2] OR “veterinary services”[Title/Abstract:~2] OR “veterinary healthcare”[Title/Abstract:~2] OR “veterinary treatment”[Title/Abstract:~2] OR “veterinary treatments”[Title/Abstract:~2]
#2	access*[Title/Abstract] OR barrier*[Title/Abstract] OR limitation*[Title/Abstract] OR facilitat*[Title/Abstract] OR challenge*[Title/Abstract]
#3	“Animal Welfare”[Mesh] OR “animal welfare” [Title/Abstract:~2] OR “animal health” [Title/Abstract:~2] OR “animal wellbeing” [Title/Abstract:~2] OR “animal well being” [Title/Abstract:~2]
#4	#1 AND #2 AND #3
#5	#4 AND LANGUAGE = ENGLISH

CAB Abstracts	
#1	DE = (veterinary services)
#2	TS = (veterinary NEAR/2 (care OR service* OR healthcare OR treatment*))
#3	#1 OR #2
#4	TS = (access* OR barrier* OR motivat* OR facilitat* OR challenge*)
#5	DE = (animal health OR animal welfare)
#6	TS = (animal* NEAR/3 (welfare OR health OR wellbeing OR “well being”))
#7	#5 OR #6
#8	#3 AND #4 AND #7
#9	#8 AND LANGUAGE = ENGLISH

Upon the removal of duplicates, two independent reviewers (KP, AD) began title/abstract screening of the first 25 publications for reliability purposes (percentage agreement = 0.8). The remaining 1,017 title/abstracts were screened by one reviewer (KP), although any doubt was discussed amongst the two reviewers (KP and AD) and resolved. The full-text screening also began with both reviewers (KP and AD) screening a subset of the first 41 publications (percentage agreement = 0.63) to establish agreement and the remaining 369 publications were screened independently (KP). Each of the full-text publications selected by KP was discussed with AD to reach unanimous agreement on the final inclusion list.

### 2.4 Data extraction and synthesis

Each of the included full-text publications had information manually extracted. The following information was noted: (1) Covidence ID, (2) decision to include (Y/N), (3) author(s), (4) publication year, (5) title, (6) journal, (7) journal topic area, (8) type of publication (e.g., research, review), (9) country where the study was conducted, (10) animal of interest, (11) definition(s) of access to veterinary care reported, (12) perceived barriers to accessing veterinary care reported (e.g., cost of veterinary services, distance to service provider, cultural competency), (13) animal health interventions reported (e.g., spay/neuter services, One Health clinics), (14) impact of access to veterinary care on animal welfare (e.g., reduced disease prevalence), and (15) key results and outcomes. Information about the journals’ subject area of research was retrieved from ‘Scimago JR’, a search engine with predefined fields, by recording each subject area of the journal. If a publication included (1) a study that was conducted in more than one country, (2) more than one focal animal, (3) more than one type of focal animal, or (4) a definition of ‘access’ that addressed more than one theme, each was identified and accounted for. The global economic status of each country represented was determined according to the World Economic Situation and Prospects Annex (31).

## 3 Results

### 3.1 Literature search and strategy

**Figure 1:**
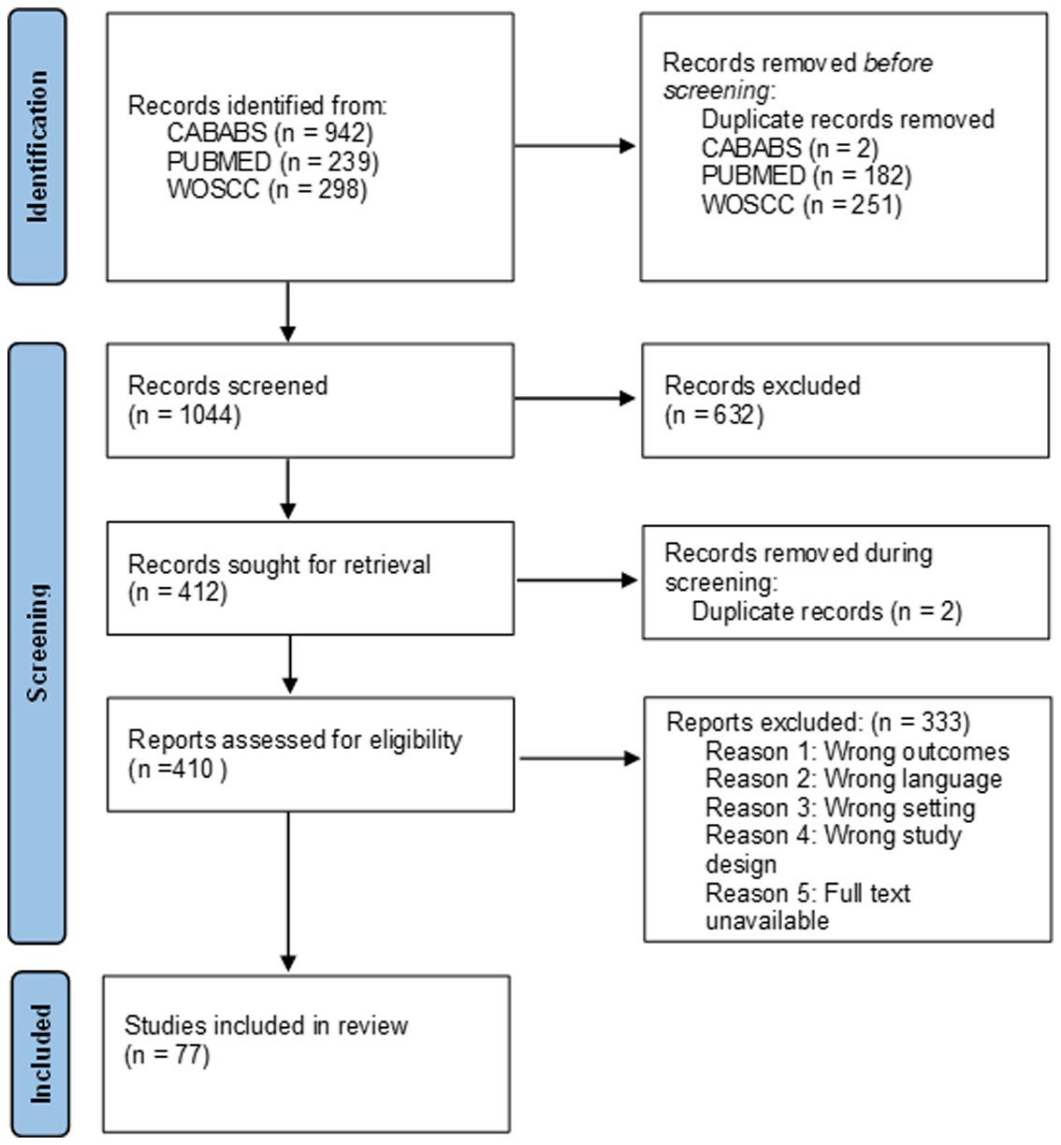
PRISMA flow diagram.

Of the 1,042 publications, 632 were considered to be non-relevant based upon their titles and abstracts and were therefore excluded, leaving 410 for full-text screening. Of the 410 full-text publications screened, 335 were excluded due to failure to meet inclusion criteria. A total of 77 publications, including 8 reviews and 69 research studies, were identified as relevant and included in the scoping review (Figure 1).

### 3.2 Characteristics of the publications

#### 3.2.1 Journal subject area

Half (n = 59, 50%) of the included publications were published in journals with “veterinary” being the primary subject area, followed by journals specializing in topics related to “agriculture” (*n* = 30, 26%) and “medicine” (*n* = 15, 13%) (Table 2).

**Table 2:** Number and percentage of publications by journal subject area.^a^

Journal subject area	*n*	%
Veterinary	59	50
Agriculture	30	26
Medicine	15	13
Multidisciplinary	6	5
Social Sciences	4	3
Business	2	2
Environmental Science	1	1
Total	117*	100

a Information about the subject area of research was retrieved from ‘Scimago JR’ (https://www.scimagojr.com/, accessed on January 2023) by recording each subject area of the journal that was predefined by the search engine. *In the case in which a journal addressed more than one topic area, each topic area was accounted for in the calculation.

#### 3.2.2 Country of study

**Figure 2:**
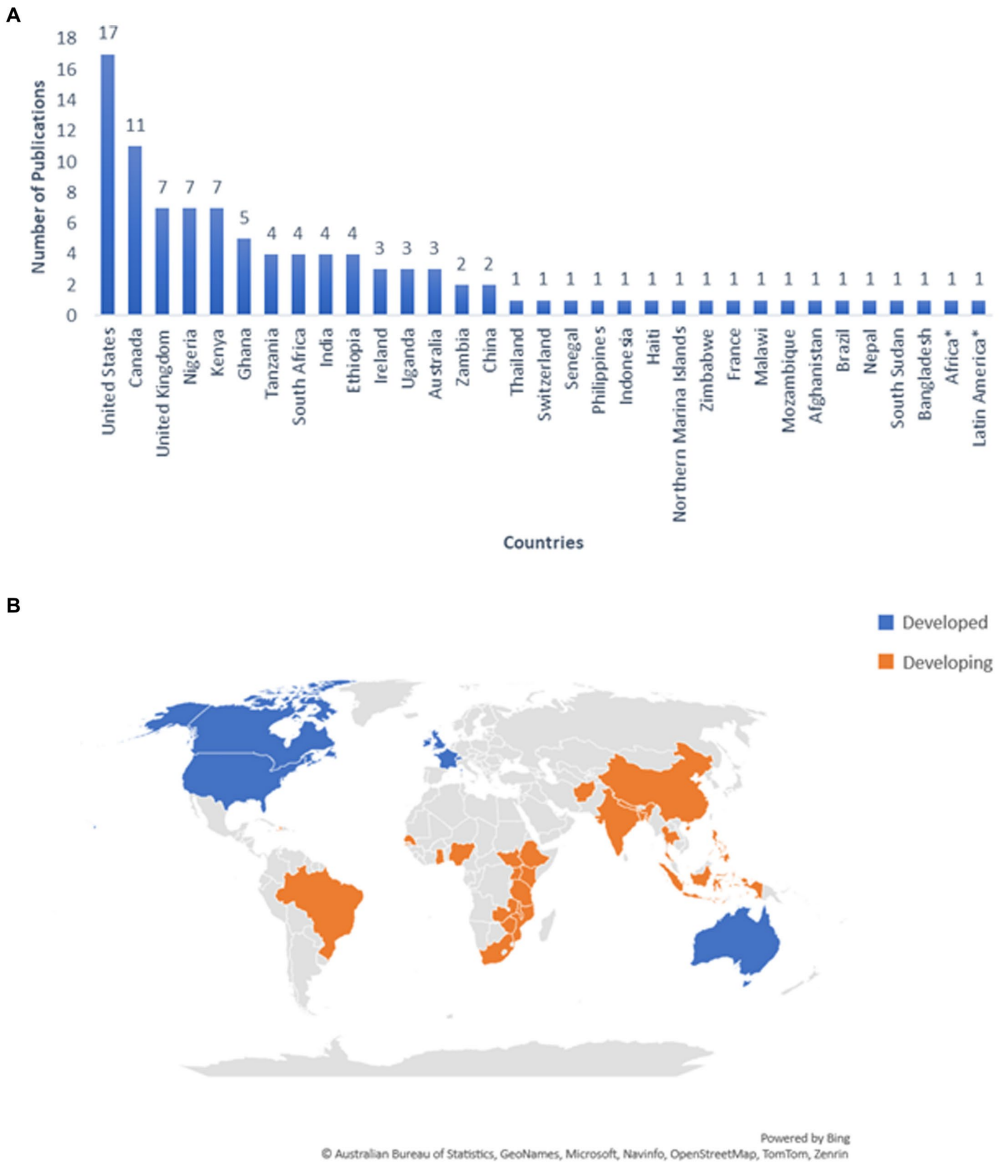
**(A)** Number of publications by country; **(B)** the economic classification of the countries represented in the literature according to the World Economic Situation and Prospects Annex (31). Some publications consisted of reviews that discussed broad geographic regions, rather than individual countries. In the case in which a study was conducted in more than one country, each country was accounted for in the calculation.

A total of 33 different countries and regions are represented in this scoping review. The countries in which studies were most frequently conducted included the United States (*n* = 17, 16.8%) and Canada (*n* = 11, 10.9%) (Figure 2A). Countries and regions were further classified based upon their global economic status and were identified as either “developed” (*n* = 7, 21%) or “developing” (*n* = 26, 79%) according to the World Economic Situation and Prospects Annex (31) (Figure 2B).

#### 3.2.3 Publication timeline

**Figure 3:**
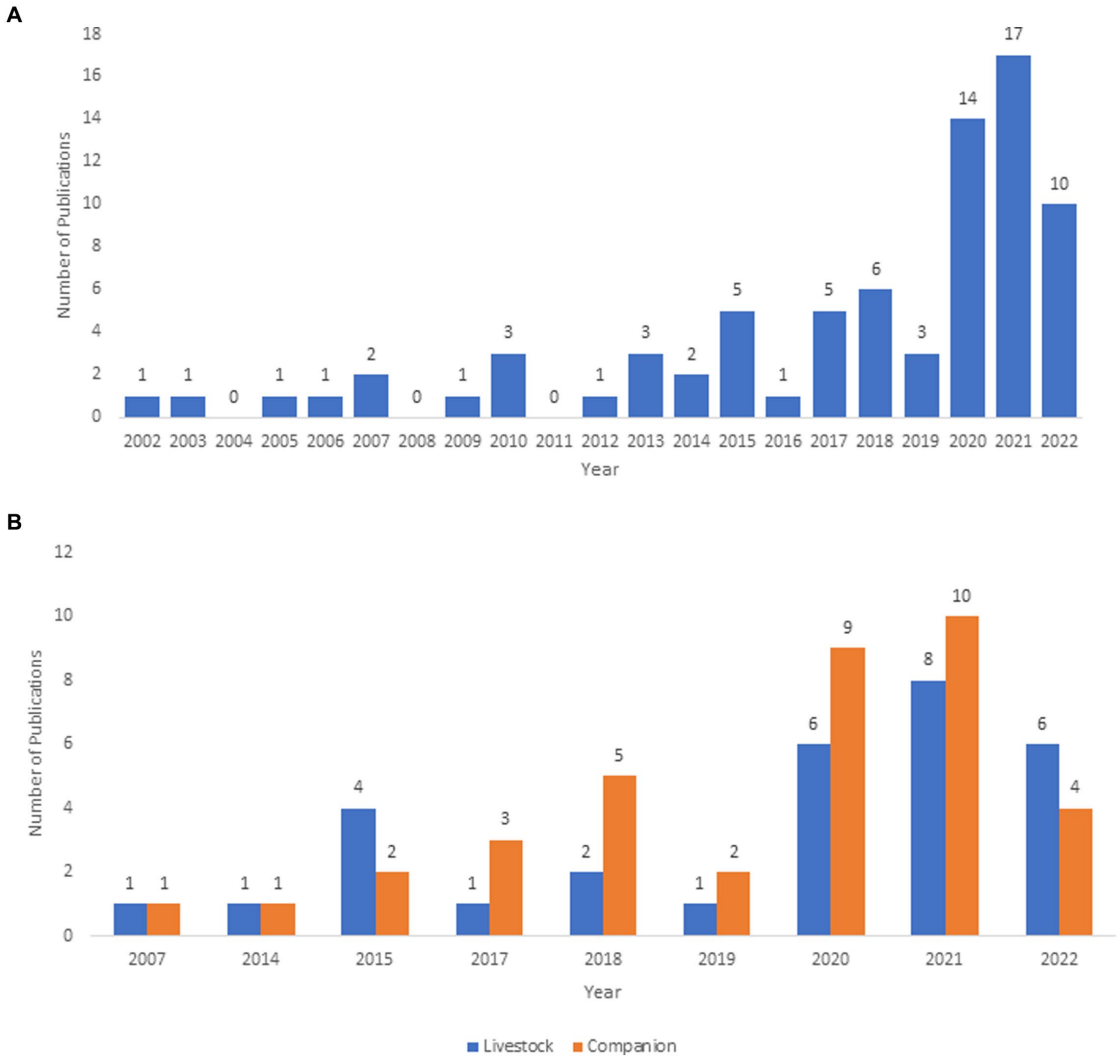
**(A)** Number of publications by year of publication; **(B)** number of publications classified according to the type of animals studied. In the case in which a publication included both livestock and companion focal animals, both groups were accounted for in the calculation. All years shown in **(B)** are those including publications having both livestock and companion as the animals of interest.

The earliest publication included in this scoping review addressed access to veterinary care for livestock species and was published in 2002 (Figure 3A). In more recent years, this topic has not only gained more attention, but it has also expanded to explore the challenges related to accessing veterinary care for both livestock and companion animals (Figure 3B). Specifically, in the years 2020 (*n* = 16, 19%) and 2021 (*n* = 19, 22%) (Figure 3A), the number of publications addressing access to veterinary care more than tripled over previous years.

#### 3.2.4 Species studied

**Figure 4:**
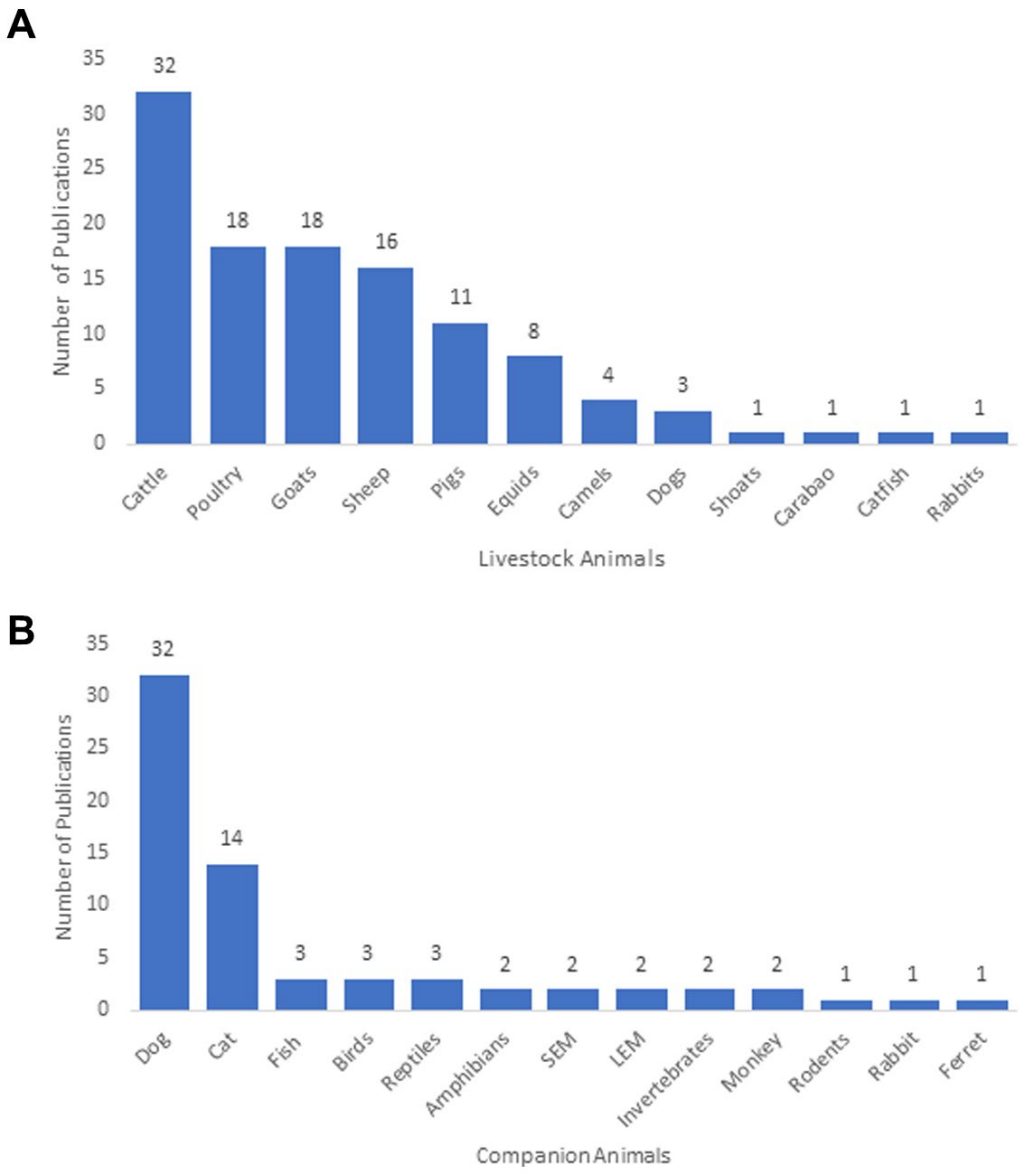
**(A)** Number of publications that studied livestock animals; **(B)** number of publications that studied companion animals. All livestock and companion animals were classified according to how they were identified in their respective publications; SE, small exotic mammal (<20 kg); LEM, large exotic mammal (>20 kg); Shoat, sheep-goat hybrid.

The types of animals studied were classified as either livestock species (*n* = 43, 54%) (e.g., used for income generation, food sources or draft purposes) or companion species (*n* = 37, 46%) (e.g., used for stress relief or companionship) according to how they were identified within their respective publications. The most frequently studied livestock and companion animals were cattle (*Bos taurus*) (*n* = 32, 28%) and dogs *(Canis familiaris)* (*n* = 32, 84%), respectively (Figure 4).

### 3.3 How access to veterinary care is broadly defined

Broad definitions of access to veterinary care were not identified in the included publications. Most publications that thoroughly discussed access to veterinary care did not provide an explicit definition for the term (*n* = 67, 87%). Where definitions were provided (*n* = 10, 13%), they were proposed by either the researchers conducting the study (*n* = 7), the animal caretakers themselves (*n* = 1), or the origin of the definition remained unclear (*n* = 2). The definitions provided for access to veterinary care were highly variable. Six different themes emerged in the definitions of access to veterinary care (Table 3). Themes included: geographical proximity (*n* = 5, 36%) (e.g., distance to the nearest veterinary service), affordability (*n* = 2, 14%) (e.g., affordability for various livestock keepers), service utilization (*n* = 2, 14%) (e.g., whether or not a farmer has used any veterinary services in the last year of production), service availability (*n* = 2, 14%) (e.g., preventative healthcare measures such as routine vaccination are available), communication (*n* = 2, 14%) (e.g., accessibility of professionals, physical and communicative, and ease of contact), and physical accessibility (*n* = 1, 7%) (e.g., good signage, clear of obstructions, and suitable handrails in appropriate locations). See Table 3 for examples of the definitions that related to the themes identified.

**Table 3:** Number of publications that defined “access to veterinary care” sorted into the 6 identified themes.^a^

Defining access to veterinary care	*n*	Examples	Citation(s)
Geographical proximity	5	Spatial accessibility to animal health care consists of the distance or time between patient location and service points.	(27, 43, 44, 62, 63)
Affordability	2	Access was defined to include the availability of a service in a location and its affordability for the various livestock keepers.	(62, 64)
Service utilization	2	Access to veterinary care was operationalized as whether or not a farmer has used any veterinary services in the last 1 yr. of pig production.	(16, 45)
Service availability	2	Access to veterinary services means preventative healthcare measures, such as routine vaccination and deworming are available.	(64, 65)
Communication	2	Accessibility is defined as geographical proximity of up-to-date resources and facilities, accessibility of professionals (physical and communicative), and ease of contact.	(27, 44)
Physical accessibility	1	Accessibility of the physical space is defined as signage with good contrast and clear directions, clear of obstructions, and suitable handrails in appropriate locations.	(66)

a In the case in which a definition addressed more than one theme, each theme was accounted for in the calculation.

### 3.4 Barriers to accessing veterinary care

Identifying and addressing the barriers associated with accessing veterinary care is heavily discussed in the published literature. Several potential barriers to accessing veterinary care were identified and sorted into the following themes: financial limitations, geographic location, limited personnel/equipment, transportation, veterinarian-client relationship, client identity, appointment availability, client mental/physical condition, government support, and the COVID-19 pandemic (Table 4). Of the 77, there were only 8 publications that did not thoroughly discuss these barriers. The most frequently reported barrier to accessing veterinary care was financial limitations (*n* = 57, 27%) followed by geographic location (*n* = 35, 16%), and limited personnel/equipment (*n* = 32, 15%) (e.g., lack of service providers, lack of medical supplies). The limited amount of research pertaining to potential barriers such as the veterinarian-client relationship (*n* = 20, 9%), client identity (*n* = 17, 8%) (e.g., gender, age, language/cultural differences), appointment availability (*n* = 14, 7%) (e.g., client scheduling), and the client’s mental/physical condition (*n* = 6, 3%) is a major gap in the literature identified in this scoping review.

**Table 4:** Number of publications that mentioned barriers to accessing veterinary care sorted into the 10 identified themes.^a^

Barriers to accessing veterinary care	*n*	Citation(s)
Financial limitations	57	(6, 7, 11, 15–17, 20, 22, 23, 26, 38, 43, 46, 47, 49, 60, 62, 64, 65, 67–104)
Geographic location	35	(7, 17, 20, 23, 26, 43, 49, 50, 58, 60, 63, 65, 67, 71, 73, 75–78, 82, 84, 85, 89, 91, 93, 96, 98, 99, 102, 103, 105–108)
Limited personnel/equipment	32	(6, 20, 26, 38, 46, 49, 50, 58, 63, 64, 69, 70, 72, 74, 76, 78, 80–82, 84, 85, 88, 90, 91, 95, 98, 102, 106, 108–111)
Transportation	24	(11, 15, 17, 20, 22, 23, 26, 45, 47, 49, 50, 60, 67, 69, 70, 72, 77, 81, 89, 92, 94, 97, 100, 103)
Veterinarian-client relationship	20	(7, 22, 23, 38, 44, 49, 66, 68, 70, 71, 78, 80, 84, 88, 89, 92, 97–99, 102)
Client identity	17	(7, 11, 20, 23, 47, 62, 65, 77, 80, 82, 92, 93, 96, 97, 99, 104, 107)
Appointment availability	14	(11, 43, 44, 60, 64, 75, 78, 81, 82, 92, 97, 100, 102, 106)
Client mental/Physical condition	6	(20, 22, 66, 81, 100, 107)
Government support	6	(15, 38, 65, 74, 88, 109)
COVID-19 pandemic	4	(22, 47, 86, 100)

a In the case in which a publication mentioned more than one barrier to accessing veterinary care, each barrier was accounted for in the calculation.

### 3.5 Impacts of access to veterinary care on animal welfare

Our understanding of how access to veterinary care may impact the welfare of animals is fairly limited with only a few (*n* = 13, 17%) of the included publications reporting on such potential impacts (Table 5). In this subset of publications, how access to veterinary care impacts the health and functioning of animals (i.e., body condition score, vaccination status, length of working life) was unanimously captured in the literature (*n* = 13, 100%). However, only two publications reported on how access to veterinary care may have an impact on behavior (*n* = 2, 15%) (i.e., frequency of barking, roaming behaviors).

**Table 5:** Number of publications that reported on the impact access to veterinary care may have on animal welfare sorted into two components of animal welfare.^a^

Welfare components	*n*	Citation(s)
Physical Health	13	(15, 57, 58, 77, 81, 89, 93, 98, 112–116)
Behavior	2	(89, 113)

a In the case in which a publication reported impacts on more than one component of animal welfare, each component was accounted for in the calculation.

## 4 Discussion

Developing solutions to improve access to veterinary care requires that we first have a thorough understanding of what is meant by the term, ‘access to veterinary care’. Not only must we have a clear definition, our understanding of the factors that potentially influence access to veterinary care and the perceptions of such access must be improved. This scoping review yielded insights into how “access” to veterinary care has been broadly defined to date which encompassed definitions in terms of geographical proximity, affordability, service utilization, service availability, communication, and physical accessibility. This allowed us to outline the potential barriers recognized in scientific literature that may influence access to veterinary care, the extent to which they are discussed in the literature, and identify how differences in access to veterinary care may impact the welfare of both companion and livestock animals.

A total of 1,044 results were retrieved from the database searches and only 77 publications were identified as relevant to the topic. This indicates that a majority of seemingly relevant publications were primarily focused on vaguely discussing the importance of accessing veterinary care and only alluded to the barriers that restrict access to care and their potential impacts on animal welfare. Although half of the included studies were published by veterinary journals (n = 59, 50%), the results of this scoping review demonstrate that access to veterinary care is a complex and interdisciplinary issue, with social, economic, and ethical implications (32). Thus, while it may seem intuitive for the veterinary community to spearhead access to care discussions, greater incorporation of multidisciplinary expertise and coverage of the subject by journals that appeal to a broader range of scholars, such as economists and other social scientists, might help to identify blind-spots and methodologies that enhance and build on the contributions of the existing literature.

The United States (*n* = 17) and Canada (*n* = 11) currently lead in the number of studies published pertaining to access to veterinary care. This was somewhat expected, especially since this review’s language criteria was limited to only include publications written in English, which is the most frequently used language in peer-reviewed journals (33). As a result, it is important to note that researchers publishing in other languages, albeit a minority among the scientific community, may potentially be producing publications related to this topic that have yet to be translated to English. Given the global impacts of access to veterinary care and the likelihood that geographical differences influence access, additional primary research in regions where information gaps currently exist should be prioritized for future research. For example, in countries such as Bangladesh where publications were scarce (*n* = 1) there is a need to further explore the potential barriers limiting access to veterinary care as well as their impacts on animal welfare that are relevant. Additionally, in countries such as Ghana where the literature was slightly more robust (*n* = 7), further investigation is needed to identify potential solutions and address the barriers to care that have already been established in the literature.

Our findings revealed that discussions of access to veterinary care have been evolving over time. For example, earlier research investigating this topic primarily focused on understanding how the issues surrounding access to veterinary care impacted livestock specie. However, in 2017, a shift began to occur, with most publications having companion animals as their species of interest. Further, there was a substantial increase in publications addressing access to veterinary care during the onset of the COVID-19 pandemic (Figure 3). The latter change may be partly due to the surging interest in companion animal acquisition and rising awareness of One Heath between 2020 and 2021 (34, 35). The concept of One Health is an integrated, unifying approach to balance and optimize the health of people, animals, and the environment. It is particularly important when attempting to prevent, predict, detect, and respond to global health threats (36). This was demonstrated in the global lockdowns experienced during the COVID-19 pandemic, where the effects of restricted access to health services for both humans and animals were amplified.

In regard to the type of focal animals featured in discussions of access to veterinary care, the most frequently studied livestock and companion animals were cattle (*n* = 32) and dogs (*n* = 32), respectively. This is likely a result of cattle being part of a billion-dollar global industry upon which many households rely for income (37). Cattle are also perceived to have significant cultural value, with many viewing them as symbols of wealth, particularly in developing countries where they are often utilized as gifts or in rituals (38). Similarly, dogs are also internationally popular due to their long history of being human companions with many households perceiving them as members of the family (1, 39). According to the American Veterinary Medical Association (AVMA) and FEDIAF (40, 41), dogs are among the most commonly kept companion animals, and over time, have been more positively perceived by the public than cats *(Felis catus)* (42). This may inadvertently create bias towards focusing on providing access to care for dogs, both by the public and in the literature.

Despite the overwhelming interest in improving access to veterinary care, the number of publications defining the term were minimal (*n* = 10) and our hypothesis that the definition of access to veterinary care would vary amongst different stakeholders (e.g., veterinarians, animal welfare organizations, animal caretakers) was generally met. Our findings indicated that even within veterinary journals, where most of the research is published, there is a lack of consistency in how access to veterinary care is defined, limited knowledge on how social barriers may impact access to veterinary care, and limited knowledge on how lack of access to veterinary care may influence the welfare of both livestock and companion animals. For example, most studies defined access to veterinary care in terms of geographical proximity (*n* = 5), with one study stating the definition of access as the “distance to the nearest veterinary service” (43). Other studies have utilized a combination of different themes to inform the definition of access to veterinary care. One study included aspects of both geographical proximity and communication to develop a definition of access which was “geographical proximity of up-to-date resources and facilities, accessibility of professionals (physical and communicative), and ease of contact” (44). In other studies, the definition of access to veterinary care was based on service utilization (n = 2) or “whether or not a farmer has used any veterinary services in the last year…” (45). This approach to defining access to veterinary care could be problematic, because, without additional context, it implies that veterinary services are only needed annually. In some cases, veterinary services are needed more than once a year to maintain health and welfare. However, it is possible they may not have been utilized due to constrained access. These variations confirm that the definitions of access to veterinary care that are currently utilized in the literature are inconsistent, and the research designed to understand the access to care issue is still in its infancy. These deficits create major challenges for veterinarians, animal scientists, and social scientists in envisioning the scope of the barriers that may be involved as well as solutions that might overcome these. There is therefore a need for further investigation prior to the development of additional initiatives intended to improve access to veterinary care.

Our second hypothesis, that access to veterinary care would differ by region, socioeconomic status, and age was also met. In addition to region, socio-economic status and age, our findings identified several other barriers that potentially impact access to veterinary care. Of the ten barriers reported to influence access to veterinary care, the most frequently reported was financial limitations (*n* = 57) which would often result in a delay in services, often causing animal health problems to become severe (46). Financial limitations are closely linked to demographic factors such as socioeconomic status, education level, and ethnic background, all of which relate to the less frequently mentioned barrier of client identity (*n* = 17). As demonstrated by Morris and colleagues (47), the challenges already faced by low-income animal caretakers when seeking veterinary care have the potential to be exacerbated by additional factors such as a perceived lack of cultural competency from their service provider. Geographic location (*n* = 35), limited personnel/equipment (*n* = 32) and transportation (*n* = 24) are additional barriers heavily discussed in the literature that could also be related to financial limitations. Further, scarcity of veterinary service providers heavily influences animal health outcomes and is often associated with residence in regions that are considered to be impoverished, rural or remote (48). This is an issue that is frequently reported in, but not unique to, animal caretakers residing in developing countries (49, 50).

Considering that economic resources shape the health and well-being of all species, within a household or production system, many underserved communities that experience human health disparities are also potentially at risk of experiencing similar disparities in veterinary healthcare systems (19, 51, 52). Many of the barriers that are understudied in regard to how they may impact access to veterinary care such as the service provider-client relationship, appointment availability, and client identity have already been identified as major barriers to accessing resources in human healthcare systems (53–56). Therefore, the disparities in accessing healthcare that are mirrored in both human and veterinary healthcare systems could potentially be explained by our limited understanding of the factors that broadly contribute to healthcare inequality. Further investigation is needed to better understand the complex inter-play between the many barriers that may contribute to health inequalities within veterinary health systems not only to safeguard animal welfare but also to improve and protect One Health.

The number of publications reporting the impacts of access to veterinary care on animal welfare were also minimal (*n* = 13), with the majority being primarily focused on physical health-related metrics (*n* = 11). However, these studies provide valuable insights about the implications of access to care for animal welfare. For example, one study implemented a community-based veterinary assistance intervention for goat producers which facilitated consistent access to veterinary services, and resulted in a reduction in the prevalence of infectious disease from 14.89% in year 1 to 6.38% in year 2 (57). Riley and colleagues (58) reported that the implementation of a companion animal health management program, which employed strategies to minimize geographical and limited personnel barriers to accessing care, not only resulted in a 77% increase in good body condition scores in dogs, but the prevalence of cats and dogs that were spayed/neutered more than doubled. Another study reported that limited access to veterinary services accompanied with substandard drug supplies were key factors contributing to persistent and frequent disease outbreaks in cattle (15). Some publications also reported the impacts of access to veterinary care on behavioral aspects of animal welfare (*n* = 2), particularly vocalizations that are often associated with nervousness or stress (59). For example, Ma and colleagues (60) found that because of efforts by the Indigenous Community Companion Animal Health Program (ICCAHP), respondents reported that the welfare of their dogs improved as evidenced by decreasing roaming and barking behaviors, improving body condition score, and improving general health.

Access to veterinary care is often associated with animal health. However, because US residents also perceive veterinarians as one of the most trusted source of animal welfare information (61), they have the potential to play a major role in providing caretakers with information on all aspects of animal welfare, including the behavioral needs of their animals. Despite being a crucial component of animal welfare, animal behavior is often overlooked in publications discussing access to veterinary care. This might be due to either lack of caretaker awareness of the importance of behavioral health, or the service provider having limited resources to support behavioral consultations. The paltry number of publications discussing the impacts of access to veterinary care on animal behavior and its relationship to welfare, combined with the overall absence of literature discussing links between access to care and animal welfare outcomes is therefore another significant gap in the literature. Future studies on access to veterinary care should further investigate the potential impacts on animal behavior and related welfare outcomes.

The findings of this scoping review yielded some conflicting results. An overwhelming number of publications discussed a limited number of potential barriers to accessing veterinary care. Yet, none established a clear, comprehensive, and consistent definition of the term. Some definitions have been proposed such as “geographical proximity of resources and service; accessibility of professionals and ease of contact” (27), “the availability of a service in a location and its affordability for various livestock keepers” (62), and “geographical proximity of up-to-date resources and facilities, accessibility of professionals (physical and communicative), and ease of contact” (44). While these offer a strong basis on which to build, each overlooks at least one important consideration that influences access to care. This suggests that different expert stakeholders hold inconsistent and possible incomplete conceptions of what the term ‘access to care’ may entail, or some may not deem it necessary to define the term in their scientific publications. This lack of consensus may constrain meeting public expectations and needs for veterinary care. Consequently, we propose the following broader and more comprehensive definition as informed by the findings of this review: *Access to veterinary care means that the economic, physical, social, mental, and emotional resources necessary for people to secure, communicate with, and benefit from the services of a trusted veterinary service provider are available as needed to optimize the health and welfare of animals in their care.* This implies that virtual or mobile services are afforded, or that brick-and-mortar veterinary facilities are within sufficiently close geographical proximity to not unduly burden clients, and that they have the necessary resources to travel to and fro, with due consideration and accommodation of those with different physical, neurological, and cognitive abilities. Access to veterinary care also requires that the services be affordable, consistently available, and delivered by personnel that are adequately trained to treat the given species and willing and able to educate the client on animal health and welfare, irrespective of their gender, ability, cultural, or socio-economic status. Achieving all of these criteria is likely to be more aspirational than fully attainable. Nevertheless, the definition proposed outlines ideal conditions veterinary professionals should strive for to increase the capacity of more members of the public to equitably secure veterinary care.

Further investigation is required to improve our understanding of the barriers surrounding access to veterinary care that are less frequently mentioned in the literature (i.e., veterinarian-client relationship, client identity, client’s mental/physical condition) and how they potentially impact all components of animal welfare. Differences in the perceptions of access to veterinary care amongst major stakeholders, such as veterinarians and the public, should also be explored in future studies.

## Data availability statement

The original contributions presented in the study are included in the article/Supplementary material, further inquiries can be directed to the corresponding author.

## Author contributions

KP: Data curation, Formal analysis, Methodology, Visualization, Writing – original draft, Writing – review & editing. AD: Methodology, Supervision, Visualization, Writing – review & editing, Data curation. JY: Data curation, Methodology, Software, Supervision, Writing – review & editing. SB: Project administration, Writing – review & editing. CC: Conceptualization, Funding acquisition, Methodology, Project administration, Supervision, Visualization, Writing – review & editing.
