# Supplementary material for: Access to veterinary care: evaluating working definitions, barriers, and implications for animal welfare
Source: Front Vet Sci. 2024 Jan 18;11:1335410. doi: 10.3389/fvets.2024.1335410 (PMC10830634; doi:10.3389/fvets.2024.1335410)
Supplement: Supplementary file 1 [file Data_Sheet_1.docx]

**Supplementary Material**

**Title: *“*****Access to Veterinary Care: Evaluating Working Definitions, Barriers, and Implications for Animal Welfare”**

Kayla Pasteur^1^, Alessia Diana^1^, Jane Kinkus Yatcilla^2^, Shanis Barnard^1^, Candace Croney^1*^

^1^Department of Comparative Pathobiology, Purdue University College of Veterinary Medicine, West Lafayette, IN, United States

^2^Purdue University Libraries, Purdue University, West Lafayette, IN, United States

*** Correspondence:** Dr. Candace Croney [ccroney@purdue.edu](mailto:ccroney@purdue.edu)

**Scoping Review Protocol *(Unpublished)***

**Relevance:**

This topic has implications for the health and welfare outcomes of both companion and livestock animals. Therefore, it is important to determine how scientific literature has defined ‘access’ to veterinary care, as well as identify the barriers influencing ‘access’ to veterinary care that have been investigated to date. Understanding how we define access to veterinary care, the barriers that constrain access, and its impact on animal health and welfare will provide insights on how to promote enhancements in the quality of veterinary interventions and improve animal welfare outcomes.

**Review Question(s)**

1. What is ‘access’ to veterinary care?
2. Who defines ‘access’ to veterinary care?
3. Who has ‘access’ to veterinary care?
4. How does ‘access’ to veterinary care impact the welfare of animals?

**Framework: PICO**

Population: Animal caretakers

Intervention: Veterinary care

Comparators/Control: Access, or lack thereof, to veterinary services

Outcome: Impacts on animal health and welfare

**Searches**

A systematic literature search will be performed using the databases PubMed, Web of Science and CAB Abstracts. Prior to initiating the database searches, a comprehensive search strategy will be developed in collaboration with a librarian and information specialist to search subject headings and keywords that relate to veterinary services (e.g. veterinary care, veterinary treatment), barriers to accessing care (e.g., access, barrier, challenge, limitation) and animal welfare (e.g. animal welfare, animal wellbeing, animal health). Publications written in English where the full text is available will be considered.

**Types of study to be included**

There is no limitation on the types of studies to be included.

**Condition or domain to be studied**

Pet-owners’ and livestock producers’ access to veterinary care services and how this access, or lack thereof, may impact the health and welfare outcomes of animals.

**Participants/population**

Inclusion criteria: (1) Publications that focus on how access to veterinary care is defined for dogs and cats that fulfill the role of companion animals; (2) Publications that examine the impacts of access to veterinary care on the welfare of dogs and cats that fulfill the role of companion animals; (3) Publications that focus on how access to veterinary care is defined for other animals (e.g., livestock, exotic pets); (4) Publications that examine the impacts of access to veterinary care on the welfare of other animals (e.g., livestock, exotic pets); (5) Publications that focus on the perception(s) of barriers surrounding access to veterinary care; (6) Publications written in English; (7) Peer-reviewed studies where the full text is available; (8) No limit on the year of publication.

Exclusion criteria: (1) Publications where the dogs and cats are studied in shelters/kennels/laboratories/zoos and do not fulfill the role of a companion animal; (2) Publications where other animals (e.g., livestock, exotic pets) are studied in shelters/kennels/laboratories/zoos; (3) Publications written in any language other than English; (4) Grey literature and publications where the full text is not available. Following the screening process, publications that appear as duplicates or do not satisfy the inclusion criteria will be removed.

**Risk of bias assessment**

To minimize bias the publication screening tool, Covidence, be utilized by both screeners during the review process. The final publications to be included will be assessed by two authors who will independently assess the publications and resolve any disagreements that may arise through discussion.

**Bibliographic & Statistical Software:** Covidence and Zotero will be utilized as the bibliographic software tools. All statistical analysis will be conducted using Excel.

**Target Audience**

Frontiers in Veterinary Science

**Type and method of review**

 Scoping review

**Supplementary Table**

| **Theme^1^** | **Category^2^** | **Citation** |
| --- | --- | --- |
| Financial Limitations | Cost of Veterinary Services  (n = 54) | [6,11,15–17,20,22,23,26,32,34,36,40–43,45–81,85] |
|  | Cost of Veterinary Medications (n = 2) | [26,44] |
|  | Client Income (n = 2) | [16,38] |
|  | Client Socio-economic Conditions (n = 2) | [7,82] |
|  | Employment/Working Conditions of Client (n =1) | [38] |
|  | Cost of Transportation (n = 1) | [53] |
|  | Lifespan of Animal (n = 1) | [68] |
| Geographic Location | Rural/Remote Region  (n = 25) | [7,20,26,32,35,36,38,43,45,49,51,55,56,60,62,63,67,69,74,76,83–87] |
|  | Distance to Veterinary Service/Medication Providers (n = 11) | [17,23,42,53,54,71,77,80,81,86,88] |
| Limited personnel/equipment | Lack of Service Providers  (n = 27) | [6,26,35,36,42,47,48,50,52,54,56,58,59,62,63,66,68,69,76,80,83,84,86,88–91] |
|  | Lack of Specialized Service Providers (n = 3) | [40,60,73] |
|  | Lack of Medical Supplies  (n = 2) | [36,89] |
|  | Incompetence of Healthcare Providers (n =2) | [44,58] |
|  | Limited Equipment Resources (n = 1) | [68] |
|  | Inadequate Healthcare Infrastructure (n = 1) | [20] |
| Transportation | Poor Access to Transportation (n = 23) | [11,15,17,20,22,23,26,39,41–43,45,47,48,55,59,67,70,72,75,78,81,83] |
|  | Poor Roads (n = 2) | [50,83] |
| Veterinary-Client Relationship | Communication Issues  (n = 9) | [7,23,27,39,46,62,66,75,80] |
|  | Lack of Awareness of How to Reach Service Providers  (n = 5) | [42,56,62,67,76] |
|  | Mistrust (n = 4) | [44,49,56,70] |
|  | Delays/cancellations in service delivery (n = 3) | [22,42,48] |
|  | Negative Previous Encounters (n = 1) | [70] |
|  | Service Providers Unwilling to Visit Clients (n = 1) | [58] |
|  | Limited Availability of Pet Health Information (n = 1) | [77] |
| Client Identity | Language/Cultural Differences (n = 5) | [11,23,55,70,77] |
|  | Cultural Competency of Service Provider (n = 4) | [11,41,74,75] |
|  | Client Education Level (n =4) | [23,38,71,87] |
|  | Gender (n = 2) | [34,87] |
|  | Client Social Status (n = 2) | [38,82] |
|  | Age (n =1) | [34] |
|  | Marital Status (n = 1) | [87] |
|  | Client Ethnicity (n = 1) | [87] |
|  | Client Social/Cultural Capital (n = 1) | [7] |
|  | Cultural Acceptance (n = 1) | [38] |
|  | Poor Attitudes of Service Providers (n = 1) | [58] |
|  | Experience/Knowledge of Owner (n = 1) | [60] |
|  | Differences in Cultural Values of Animals (n = 1) | [20] |
| Appointment Availability | Time Spent Seeking Veterinary Services (n = 8) | [32,36,43,53,56,59,60,86] |
|  | Client Scheduling (n = 3) | [33,78,80] |
|  | Service Provider Hours of Operation (n = 3) | [11,70,75] |
| Client’s Mental/Physical Condition | Client Disability (n = 2) | [22,39] |
|  | Lack of Mental/Emotional Resources (n = 2) | [20,59] |
|  | Physical Ability (n = 1) | [87] |
|  | Excessive Stress (n = 1) | [78] |
|  | Grief (n = 1) | [78] |
|  | Lack of Disability Accommodations (n =1) | [39] |
| Government Support | Lack of Government Support (n = 2) | [66,89] |
|  | Government Funding (n = 1) | [52] |
|  | Political Governance (n = 1) | [38] |
|  | Bureaucratic Difficulties  (n = 1) | [15] |
|  | State/Local Policy (n = 1) | [44] |
| COVID-19 Pandemic | Restricted Hours (n = 3) | [22,41,78] |
|  | Pandemic Related Fears  (n = 1) | [78] |
|  | Social Distancing (n = 1) | [64] |

^1^Each theme is an overarching barrier consisting of multiple categories of barriers that were identified and sorted according to common challenges and keywords. ^2^Each category is a specific barrier identified and extracted during the full text screening process.
